# Cheap Child Labour

**Published:** 1920-01-24

**Authors:** 


### Cheap Child Labour.

A disagreeable aspect of the problem of domestic service was disclosed at a meeting of the Merthyr Board of Guardians. Applications were received for children between the ages of ten and twelve years from the Children's Homes, for adoption, and several members protested against acceding to these requests on the grounds that there were many people anxious to secure young children brought up in the homes because they became useful when they were fourteen or fifteen years old. Such people took them as adopted girls, but they were really looking for cheap servants, or for a chance of getting some revenue from them. It was stated that in the past the Board had had to take children back because their lives were being sapped away. The Board took the humane and sensible view of their duty in deciding that the proper course was to keep the girls until they were of proper age and, then to find them suitable occupations at reasonable wages. Those who are anxious to adopt children generally desire them at an earlier age than ten or twelve, and in all cases, even where it seems obvious that adopted children will benefit by being taken into private homes to be cared for, it is important that the authorities who are primarily responsible for them should continue to keep an eye on their welfare. The alertness of the Merthyr Guardians ought to be brought to the notice of all other Boards whose organisation includes children's homes.

